# Editorial: Infections, non-communicable diseases, and reproductive health issues in a world beset by conflict and climate change

**DOI:** 10.4314/ahs.v23i4.1

**Published:** 2023-12

**Authors:** James K Tumwine

Welcome to this December issue of African Health Sciences in which we have selected for you papers on infectious diseases, non-communicable diseases, and sexual reproductive health issues.

Infectious diseases covered in this treatise include tuberculosis^1^-^6^; COVID-19^7^-^18^; and others including bacterial, fungal and viral illnesses^19^-^26^.

Communicable diseases, on the other hand, cover breast cancer^27^-^29^, hypertension^30^-^33^, diabetes mellitus^34^-^36^ and others. Others cover a host of subjects ranging from goiter^37^, alloimmunization^38^, kidney diseases^39^ and trauma^40^. The next set of papers, is on sexual reproductive health^41^-^52^. The rest are on nutrition^53^-^55^, mental health^56^-^58^ and a couple of hybrid issues^59^-^62^.

This issue is being released when the international climate change conference (COP28) is being held in the Middle East which, paradoxically, is a theater of un precedented conflict. One hopes that the goodwill shown at COP28 can permeate through our planet earth ushering in peace and common sense as mother earth tinkers on the verge of catastrophic extinction. We wish you fruitful reading and a peaceful festive season.
